# A New and Efficient Method for the Synthesis of 3,4-Disubstituted Pyrrolidine-2,5-diones

**DOI:** 10.3390/molecules17054936

**Published:** 2012-04-30

**Authors:** Eleonora D. Ilieva, Nevena I. Petkova, Rositca D. Nikolova

**Affiliations:** Sofia University St. Kliment Ohridski, Faculty of Chemistry and Pharmacy, 1, J. Bourchier Blvd., Sofia 1126, Bulgaria; Email: ilieva.eleonora@gmail.com (E.D.I.)

**Keywords:** pyrrolidine-2,5-dione, pyrrolidine, 3-substituted coumarins, 2-oxo-2*H*-1-benzopyrane, Michael addition reaction, rearrangement, Nef reaction

## Abstract

A newly found reaction for the synthesis of 3,4-disubstituted 1-hydroxy-pyrrolidine-2,5-diones from 3-substituted coumarins and nitromethane has been elaborated. The reaction involved a simple and convenient experimental procedure. The applicability of the rearrangement reaction is determined.

## 1. Introduction

Owing to their wide range of biological activities and pharmacological properties [1,2,3,4,5,6,7,8,9,10,11,12,13] the synthesis of substituted pyrrolidine-2,5-diones has become a field of increasing interest in organic synthesis during the last few decades. The approach based on nucleophilic acyl substitution involving 1,4-dicarboxylic acids [3,4,5,6,14] or their derivatives such as succinic anhydrides [7,8,9,14,15,16,9,14] with nucleophiles, e.g., amines or amides, is one of the most used methods for preparing pyrrolidine-2,5-diones. Other commonly used derivatives of 1,4-dicarboxylic acids are the amides [11,12,13,17,18,19,13,17], in these cases the nucleophilic acyl substitution is an intramolecular process. Some rearrangements which yield pyrrolidine-2,5-diones are also reported [20,21,22,23]. In this paper we report a simple and effective solvent free room temperature procedure for the synthesis of 3,4-disubstituted 1-hydroxypyrrolidine-2,5-diones from 3-substituted coumarins.

## 2. Results and Discussion

**Scheme 1:**
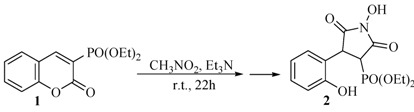
Rearrangement reaction of 3-phosphonocoumarin.

Recently, a new rearrangement reaction was reported [24] (Scheme 1) in which 3-phosphonocoumarin **1** has been transformed into the new product 1-hydroxy-4-(2′-hydroxyphenyl)-2,5-dioxopyrrolidine-3-yl-phosphonate (**2**) in excellent yield.

It is well known that numerous derivatives with anticonvulsant activity contain five- or six- membered heterocyclic rings, one or two carbonyl groups, as well as an aromatic system [25,26,27,28]. Following these findings, our attention has been focused on the preparation of 3,4-disubstituted pyrrolidine derivatives from 3-substituted coumarins to establish the limits of the rearrangement. For these purposes the reaction was carried out with coumarins bearing electron withdrawing or electron donating substituents.

### 2.1. Rearrangement Reaction of Coumarins with Electron Withdrawing Substituent in the Third Position

**Scheme 2:**
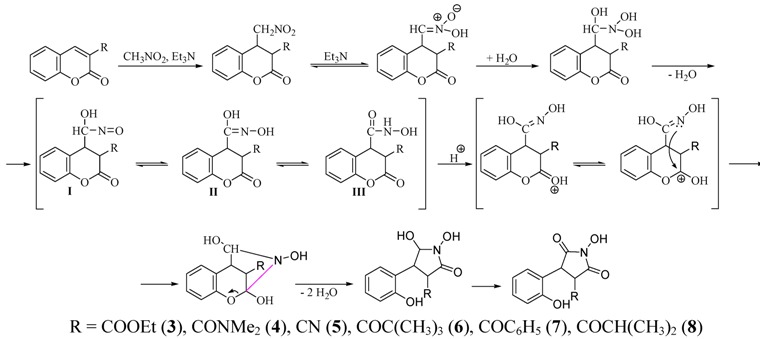
Assumed mechanism for preparation of substituted pyrrolidine-2,5-diones.

Firstly the reaction was carried out with some derivatives of coumarin-3-carboxylic acid: ethyl ester, dimethyl amide and nitrile. The corresponding pyrrolidine derivatives **3**, **4** and **5** were thus prepared (Scheme 2, Table 1). 

In these investigations the best reaction conditions for the preparation of pyrrolidines were applied [24]. It was surprising that in the case of 2-oxo-2*H*-chromene-3-carbonitrile 45% of the substrate was recovered and the yield of pyrrolidine derivative was only 17%. The reaction time was prolonged for 68 hours and the yield of the product **5** was increased to 34%, but the starting coumarin was still not consumed. When the reaction was carried out for a longer reaction time (190 hours), the reaction mixture was complicated - additional by-products were detected. It was possible to isolate only 10% of the pyrrolidine derivative **5**.

**Table 1:** Rearrangement reactions of 3-substituted coumarins.

Compound		Time, h	Yield, %
**3**	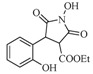	22	70%
**4**	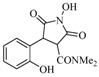	18	69%
**5**	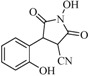	68	34%
**6**	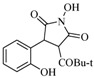	18	62%
**7**	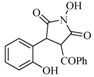	115	52%
**8**	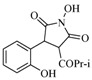	120	49%
**10**	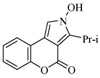		22%
**11**	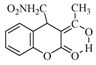	46	75%
**12**	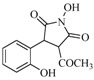		11%
**13**	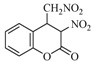	20	90%
**16**	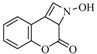	480	55%
**17**	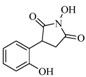	120	21%

Continuing the investigation of the studied reaction with other 3-substituted coumarins, we decided to use a series of ketones: 3-pivaloyl-, 3-benzoyl-, 3-isobutyryl- and 3-acetyl-2*H*-chromen-2-one. When the reactions were carried out with 3-pivaloyl-, 3-benzoyl- and 3-isobutyrylcoumarin (Scheme 2) the respective pyrrolidine derivatives **6**, **7** and **8** were isolated (Table 1) in good yields (62%, 52% and 49%, respectively). The corresponding reaction times were 18, 115 and 120 hours (TLC monitoring).

**Scheme 3:**
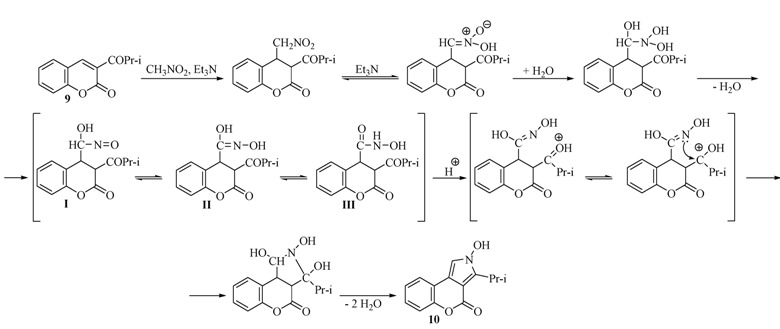
Assumed mechanism for preparation of compound **10**.

It was interesting that in the case with 3-isobutyrylcoumarin a new by-product—2-hydroxy-3-isopropylchromeno[3,4-c]pyrrol-4(2*H*)-one (**10**)—was isolated for the first time (Table 1, Scheme 3). Its formation could be explained with the easier enolization in this ketone in comparison to the other chosen ketones. In our previous work [24] formation of an isomeric structure **I** was proposed (Scheme 3) and an attack of the nitrogen atom onto the carbonyl group from the lactone ring. Now, it was assumed that there are two possible paths in the course of the reaction which would explain the formation of the by-product in the presence of a substituent providing a second carbonyl group. Thus, it was supposed that the nitrogen atom of the isomeric structure **II** is performing the attack, so formation of a new pyrrole ring is observed.

**Figure 1:**
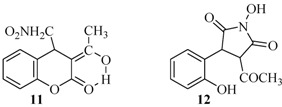
Chemical structures of compounds **11** and **12**.

The behaviour of 3-acetyl-2*H*-chromen-2-one under the studied reaction conditions was not surprising to us. It is well known that this coumarin sometimes shows a different behaviour than usually expected [29,30]. A product of Michael addition of nitromethane to the coumarin in its enol form **11** was isolated, together with pyrrolidine **12**. Products in their enol forms were isolated previously when the Michael reaction of 3-acetylcoumarin with other nucleophiles was conducted [36]. Luckily, in the present case an easy procedure for separation of the products was devised which was based on the different solubility of the compounds. A fractional extraction was applied and the product **11** (Table 1) was isolated from the methylene chloride phase. The pyrrolidine derivative **12** (Table 1) was isolated with low yield of 11% (Figure 1) from the ethyl acetate layer.

**Scheme 4:**
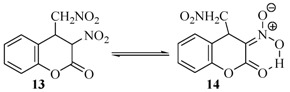
Nitro - aci-nitro tautomerism.

The reaction was also carried out with 3-nitro-2*H*-chromen-2-one, whereby compound **13**, the product of Michael reaction addition of nitromethane was isolated (Table 1). When compound **13** is in a DMSO solution it turns into its enol form **14** (Scheme 4). This fact was established from the corresponding NMR spectrum. A second spectrum of the solution was recorded a week later which showed that compound **13** was converted into **14**.

It is obvious that in contrast to the other 3-substituted coumarins, 3-acetyl-2*H*-chromen-2-one and 3-nitro-2*H*-chromen-2-one preferred to generate stable enol forms, which prevent further rearrangements. This fact explains the low yield of pyrrolidine **12** while 3-nitrocoumarin did not give any pyrrolidine at all.

**Scheme 5:**
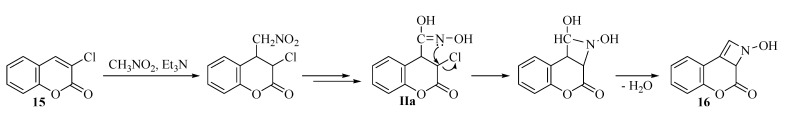
Assumed mechanism for preparation of compound **16**.

The rearrangement reaction was also carried out with 3-chloro-2*H*-chromen-2-one (**15**). A pyrrolidine derivative was not detected but a new product—2-hydroxy-2,2a-dihydrochromeno[3,4-b]azet-3-one (**16**)—was isolated, which was not observed before (Table 1, Scheme 5). In this case the assumption that the reaction mechanism goes though formation of the intermediate structure **IIa** and further attack of the nitrogen atom to C3 resulting in new azete ring seems also to be valid.

### 2.2. Rearrangement Reactions of Coumarin without a Substituent in the 3-Position

**Figure 2:**
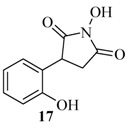
Chemical structure of compound **17**.

The investigations were continued with a study on the behaviour of 2*H*-chromen-2-one. In comparison with the previous coumarins in this case the rearrangement reaction required a long time. The pyrrolidine **17** (Figure 2) was isolated in a low yield of 21% (Table 1).

### 2.3. Rearrangement Reactions of Coumarins with Electron Donating Substituents in the 3-Position

**Scheme 6:**
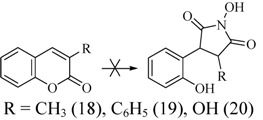
Rearrangement reaction of coumarins with electron donating substituents in the 3-position.

To check the influence of an electron donating substituent at the 3-position the reaction was carried out with 3-methyl- **18**, 3-phenyl- **19** and 3-hydroxy-2*H*-chromen-2-one **20**. No change of the starting compounds was detected (Scheme 6).

Finally, the limits of the rearrangement reaction of 3-substituted coumarins under the studied reaction conditions were established. It is noticeable that coumarins which contain electron withdrawing groups give the expected pyrrolidine derivatives. The exception to the rule are species tending to form a stable enol, whereas coumarins substituted with electron donating groups do not react at all. The absence of substituent requires a longer reaction time and decreases the yield of the product.

It is noteworthy that the developed method is accomplished by a simple and convenient experimental procedure. The products could be isolated by fractional extraction. It was noted that the pyrrolidine derivatives are more soluble in ethyl acetate, whereas the Michael products as well as the other by-products are soluble in methylene chloride. In the course of the present work it could be concluded that our investigations expand the scope of the known rearrangements of 3-substituted coumarins. The mechanism of the studied rearrangement involves different reaction paths depending on the electronic properties and the type of functional group in the 3-position of the coumarin. In addition, these investigations confirmed our suggestion on the isomeric intermediate formation. The formed tautomeric structures contribute to the rearrangement reaction.

**Figure 3:**
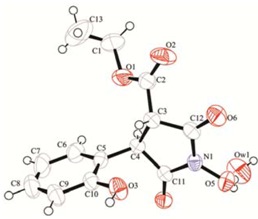
X-ray crystallographic structure of **3**.

**Figure 4:**
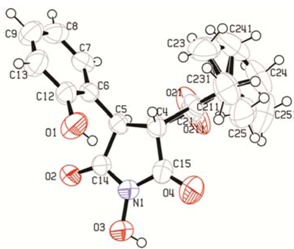
X-ray crystallographic structure of **6**.

A very important advantage of the newly found rearrangement is that it is a stereospecific reaction. All measured coupling constants for the protons 3-H and 4-H are in the range of 3.6 to 4.8 Hz which suggests an anticlinal disposition between the two atoms and is in accordance with the crystal structure for compounds **2** [24], **3** and **6** (Figure 3 and Figure 4).

## 3. Experimental

### 3.1. General

Melting points were determined with a Kofler hot-stage apparatus and are uncorrected. Infrared (IR) spectra were recorded with a Specord IR 75 spectrophotometer. ^1^Н-NMR, ^13^С-NMR and ^31^P-NMR spectra were recorded on a Bruker Advance DRX 250 (at 250 MHz for ^1^Н, 62.9 MHz for ^13^C and 101.3 MHz for ^31^P respectively) or Bruker Advance II+ 600 (at 600 MHz for ^1^Н, 150.9 MHz for ^13^C and 242.9 MHz for ^31^P respectively) spectrometer. Chemical shifts are given in ppm downfield from tetramethylsilane as the internal standard in CDCl_3_ or DMSO; coupling constants are given in Hz. The X-ray diffraction intensities were measured on a Bruker Smart X2S diffractometer, using microsource Mo-K_α_ radiation and employing the ω scan mode. The data were corrected for Lorentz and Polarization effects. An absorption correction is based on multiple scanned reflections. The crystal structures were solved by direct methods using SHELXS-97. The crystal structures were refined by full-matrix least-squares refinement against F^2^. Anisotropic displacement parameters were introduced for all non-hydrogen atoms. The hydrogen atoms attached to carbon were placed at calculated positions and refined allowing them to ride on the parent carbon atom. The hydrogen atoms bound to nitrogen and the oxygen were constrained to the positions which were confirmed from the difference map and refined with the appropriate riding model, which the exception of the amino and water hydrogen atoms. E.I. mass spectra were obtained at Thermo Scientific DFS High Resolution Magnetic Sector MS. Microanalyses were performed on VarioEL III CHNS/O, Elementar Analysen Systeme GmbH. Column chromatography was carried out on silica gel (Merck 0.063–0.2 mm and 0.043–0.063 mm) using as eluent *n*-hexane/EtOAc mixtures with increasing polarity. The reactions were monitored by TLC on silica gel 60 F_254_. All chemical reagents were purchased from Merck, Fluka and Aldrich. The starting diethyl ester of coumarin-3-phosphonic acid, ethyl ester of coumarin-3-carboxylic acid, dimethyl amide of coumarin-3-carboxylic acid, 2-oxo-2*H*-chromene-3-carbonitrile, 3-pivaloyl-2*H*-chromen-2-one, 3-isobutyryl-2*H*-chromen-2-one, 3-benzoyl-2*H*-chromen-2-one, 3-acetyl-2*H*-chromen-2-one, 3-nitro-2*H*-chromen-2-one, 3-methyl-2*H*-chromen-2-one and 3-phenyl-2*H*-chromen-2-one were prepared according to the described procedures [31,32,33,34,35,36,37,38,39].

### 3.2. General Procedure

To the starting 3-substituted coumarin (2 mmol) was added nitromethane (2 mL) and 2 mmol of the base—Et_3_N. The solution was left at room temperature until the starting materials were consumed (TLC monitoring). The reaction mixture was poured into hydrochloric acid (2 N, 4 mL) and ice, and a fractional extraction was applied—first with dichloromethane and secondly with ethyl acetate and the organic solutions were dried with anhydrous sodium sulphate. The products were purified by column chromatography (*n*-hexane and ethyl acetate mixtures of increasing polarity) or recrystallization (ethanol). 

*Ethyl 1-hydroxy-4-(2-hydroxyphenyl)-2,5-dioxopyrrolidine-3-carboxylate *(**3**).Yield: 0.317 g (70%); pale yellow crystals, purified by column chromatography on silica gel (hexane/ethyl acetate, 9:1 to 1:1), m.p. = 88–90 °C. IR (nujol): ν = 3600, 3480, 3270, 1720, 1700, 1590, 1450, 1370 cm^−1^; ^1^H-NMR (600 MHz, DMSO-d_6_): δ = 1.21 (t, ^3^*J*_HH_ = 6.9 Hz, ^3^*J*_HH_ = 6.9 Hz, 3H, CH_3_), 3.80 (d, ^3^*J*_HH_ = 5.1 Hz, 1H, 3-CH), 4.19 (q, ^2^*J*_HH_ = 14.4 Hz; ^3^*J*_HH_ = 7.2 Hz, 2H, CH_2_), 4.27 (d, ^3^*J*_HH_ = 5.1 Hz,1H, 4-CH), 6.79–6.83 (m, 2H, 5′-CH, 3′-CH), 7.17–7.24 (m, 2H, 4′-CH, 6′-CH), 10.05 (s, 1H, OH), 11.13 (s, 1H, N-OH); ^13^C-NMR (150.9 MHz, DMSO-d_6_): δ = 14.4 (s, CH_3_), 45.0 (s, CH-4), 51.0 (s, CH-3), 62.3 (s, CH_2_), 115.8 (s, CH-5′), 119.6 (s, CH-3′), 122.8 (s, C-1′), 130.0 (s, CH-6′), 131.9 (s, C-4′), 155.5 (s, C-2′), 167.8 (s, C=O), 168.1 (s, C=O), 172.4 (s, C=OOCH_2_CH_3_); MS *m/z* (%): 279 (M^+^) (18), 233 (15), 206 (18), 173 (94), 149 (100), 121 (10); Anal. Calcd. for C_1__3_H_1__3_NO_6_ (279.25): C 55.91; H 4.69; N 5.02%. Found: C 55.94; H 4.65; N 5.03%. X-ray crystallography: tetragonal, space group I4(1)cd, a 24.715(2) b 24.715(2) c 9.2853(8) Å, α 90.00 β 90.00γ 90.00, V = 5671.8(9), μ = 0.115 mm^−1^, F(000) = 2496.

*1-Hydroxy-4-(2-hydroxyphenyl)-N,N-dimethyl-2,5-dioxopyrrolidine-3-carboxamide *(**4**). Yield: 0.395 g (69%); colorless crystals, purified by recrystallization from ethanol, m.p. = 164–165 °C. IR (nujol): ν = 3180, 1720, 1700, 1620, 1480, 1380 cm^−1^; ^1^H-NMR (600 MHz, DMSO-d_6_): δ = 2.89 (s, 3H, CH_3_), 2.98 (s, 3H, CH_3_), 4.25 (d, ^3^*J*_HH_ = 3.6 Hz, 1H, 3-CH), 4.32 (d, ^3^*J*_HH_ = 3.6 Hz, 1H, 4-CH), 6.76–6.83 (m, 2H, 5′-CH, 3′-CH), 7.15–7.18 (m, 2H, 4′-CH, 6′-CH), 10.00 (s, 1H, OH), 11.00 (s, 1H, N-OH); ^13^C-NMR (150.9 MHz, DMSO-d_6_): δ = 35.6 (s, CH_3_), 37.4 (s, CH_3_), 44.7 (s, CH-4), 47.7 (s, CH-3), 115.4 (s, CH-5′), 119.1 (s, CH-3′), 122.7 (s, C-1′), 129.3 (s, CH-6′), 131.2 (s, C-4′), 155.1 (s, C-2′), 166.4 (s, C=O), 169.0 (s, C=O), 172.7 (s, C=O(CON(CH_3_)_2_); MS *m/z* (%): 278 (M^+^) (14), 218 (42), 215 (50), 206 (22), 173 (100), 146 (52), 131 (14), 118 (28), 115 (35); Anal. Calcd. for C_1__3_H_1__4_N_2_O_5_ (278.26): C 56.11; H 5.07; N 10.07%. Found: C 56.13; H 5.10; N 10.06%.

*1-Hydroxy-4-(2-hydroxyphenyl)-2,5-dioxopyrrolidine-3-carbonitrile *(**5**). Yield: 0.156 g (34%); yellow crystals, purified by column chromatography on silica gel (hexane/ethyl acetate, 9:1 to 1:1), m.p. = 128–131 °C. IR (nujol): ν = 3300, 2250, 1780, 1720, 1700, 1600, 1550, 1450, 1380 cm^−1^; ^1^H-NMR (600 MHz, DMSO-d_6_): 4.50 (d, ^3^*J*_HH_ = 4.1 Hz, 1H, 3-CH), 5.07 (d, ^3^*J*_HH_ = 4.1 Hz, 1H, 4-CH), 6.80–6.86 (m, 2H, 5′-CH, 3′-CH), 7.19–7.26 (m, 2H, 4′-CH, 6′-CH), 10.24 (s, 1H, OH), 10.78 (s, 1H, N-OH); ^13^C-NMR (62.9 MHz, DMSO-d_6_): δ = 45.1 (s, CH-4), 53.8 (s, CH-3), 110.6 (s, CN), 115.3 (s, CH-5′), 119.1 (s, CH-3′), 120.5 (s, C-1′), 129.9 (s, CH-6′), 130.6 (s, C-4′), 154.7 (s, C-2′), 155.3 (s, C=O), 168.3 (s, C=O); MS *m/z* (%): 232 (M+) (5), 175 (92), 171 (32), 147 (40), 146 (100), 130 (32), 103 (12), 91 (26); Anal. Calcd. for C_1__1_H_8_N_2_O_4_ (232.19): C 56.90; H 3.47; N 12.06%. Found: C 56.90; H 3.45; N 12.03%.

*1-Hydroxy-3-(2-hydroxyphenyl)-4-pivaloylpyrrolidine-2,5-dione* (**6**). Yield: 0.360 g (62%); colorless crystals, purified by column chromatography on silica gel (hexane/ethyl acetate, 9:1 to 1:1), m.p. = 205–206 °C. IR (nujol): ν = 3420, 3180, 1700, 1680, 1470, 1380 cm^−1^; ^1^H-NMR (600 MHz, DMSO-d_6_): δ = 1.00 (s, 9H, C(CH_3_)_3_), 4.00 (d, ^3^*J*_HH_ = 4.5 Hz, 1H, 3-CH), 4.38 (d, ^3^*J*_HH_ = 4.5 Hz, 1H, 4-CH), 6.76–6.83 (m, 2H, 5′-CH, 3′-CH), 7.15–7.20 (m, 2H, 4′-CH, 6′-CH), 10.00 (s, 1H, OH), 11.05 (s, 1H, N-OH); ^13^C-NMR (150.9 MHz, DMSO-d_6_): δ = 24.7 (s, CH_3_), 44.3 (s, C(CH_3_)_3_), 45.9 (s, CH-4), 51.4 (s, CH-3), 115.3 (s, CH-5′), 119.1 (s, CH-3′), 122.5 (s, C-1′), 129.4 (s, CH-6′), 131.3 (s, C-4′), 155.0 (s, C-2′), 169.4 (s, C=O), 172.8 (s, C=O), 211.1 (s, C=O(CO(CH_3_)_3_); MS *m/z* (%): 291 (M^+^) (16), 206 (26), 201 (28), 190 (23),174 (53), 146 (100), 118 (34), 115 (12); Anal. Calcd. for C_1__5_H_1__7_NO_5_ (291.30): C 61.85; H 5.88; N 4.81%. Found: C 61.81; H 5.85; N 4.79%. X-ray crystallography: monoclinic, space group P 21/c, a 15.3654(15) b 9.7072(9) c 10.4846(10) Å, α 90.00 β 106.019(3) γ 90.00, V = 1503.1(2), μ = 0.097 mm^−1^, F(000) = 616.

*3-Benzoyl-1-hydroxy-4-(2-hydroxyphenyl)pyrrolidine-2,5-dione *(**7**). Yield: 0.321 g (52%); colorless crystals, purified by column chromatography on silica gel (hexane/ethyl acetate, 9:1 to 1:1), m.p. = 185–185.6 °C. IR (nujol): ν = 3400, 3100, 1690, 1680, 1600, 1500, 750, 680 cm^−1^; ^1^H-NMR (600 MHz, DMSO-d_6_): δ = 4.43 (d, ^3^*J*_HH_ = 4.2 Hz, 1H, 3-CH), 5.03 (d, ^3^*J*_HH_ = 4.2 Hz,1H, 4-CH), 6.75–6.85 (m, 2H, 5′-CH, 3′-CH), 7.13–7.19 (m, 2H, 4′-CH, 6′-CH), 7.53–7.55 (m, 2H, 2′′-CH, 6′′-CH), 7.70 (t, ^3^*J*_HH_ = 7.5 Hz, 1H, 4′′-CH), 7.95–7.97 (m, 2H, 3′′-CH, 5′′-CH), 10.06 (s, 1H, OH), 11.11 (s, 1H, N-OH); ^13^C-NMR (150.9 MHz, DMSO-d_6_): δ = 43.8 (s, CH-4), 52.7 (s, CH-3), 115.3 (s, CH-5′), 119.0 (s, CH-3′), 122.6 (s, C-1′), 128.8 (s, CH-2′′,CH-6′′), 129.3 (s, CH-1′′), 129.4 (s, CH-3′′,CH-5′′), 130.9 (s, CH-6′), 134.4 (s, C-4′′), 135.2 (s, CH-4′), 155.2 (s, C-2′), 168.3 (s, C=O), 172.4 (s, C=O), 194.0 (s, C=O(COC_6_H_5_)); MS *m/z* (%): 311 (M^+^) (27),293 (18), 277 (34),276 (49), 250 (78), 234 (34), 221 (64), 207 (42), 206 (100), 173 (77), 149 (42), 147 (90); Anal. Calcd. for C_17_H_13_NO_5_ (311.29): C 65.59; H 4.21; N 4.50%. Found: C 65.55; H 4.23; N 4.53%.

*1-Hydroxy-3-(2-hydroxyphenyl)-4-isobutyrylpyrrolidine-2,5-dione *(**8**). Yield: 0.269 g (49%); yellow crystals, purified by column chromatography on silica gel (hexane/ethyl acetate, 9:1 to 1:1), m.p. = 160–162 °C. IR (nujol): ν = 3400, 3180, 1720, 1680, 1470, 1340, 1380 cm^−1^; ^1^H-NMR (600 MHz, DMSO-d_6_): δ = 0.97 (d, ^3^*J*_HH_ = 6.6 Hz, 3H, CH_3_), 0.99 (d, ^3^*J*_HH_ = 7.2 Hz, 3H, CH_3_), 2.94–2.98 (m, 1H, CH(CH_3_)_2_), 4.18 (d, ^3^*J*_HH_ = 4.8 Hz, 1H, 3-CH), 4.23 (d, ^3^*J*_HH_ = 4.8 Hz, 1H, 4-CH), 6.76–6.82 (m, 2H, 5′-CH, 3′-CH), 7.15–7.18 (m, 2H, 4′-CH, 6′-CH), 9.98 (s, 1H, OH), 11.00 (s, 1H, N-OH); ^13^C-NMR (150.9 MHz, DMSO-d_6_): δ = 17.0 (s, CH_3_), 17.6 (s, CH_3_), 40.4 (s, CH(CH_3_)_2_), 43.5 (s, CH-4), 54.9 (s, CH-3), 115.3 (s, CH-5′), 119.1 (s, CH-3′), 122.8 (s, C-1′), 129.3 (s, CH-6′), 131.1 (s, C-4′), 155.1 (s, C-2′), 168.4 (s, C=O), 172.4 (s, C=O), 207.8 (s, C=O(COCH(CH_3_)_2_); MS *m/z* (%): 277 (M^+^) (12), 241 (40), 226 (45), 216 (41), 207 (30), 206 (19), 173 (100), 151 (28), 148 (48),146 (48), 133 (32), 125 (68); Anal. Calcd. for C_14_H_15_NO_5_ (277.27): C 60.64; H 5.45; N 5.05%. Found: C 60.60; H 5.40; N 5.03%.

*2-Hydroxy-3-isopropylchromeno[3,4-c]pyrrol-4(2H)-one *(**10**). Yield: 0.107 g (22%); yellow crystals, purified by column chromatography on silica gel (hexane/ethyl acetate, 9:1 to 1:1), m.p. = 85–88 °C. IR (nujol): ν = 3250, 1730, 1680, 1620, 1460, 1400, 1380 cm^−1^; ^1^H-NMR (600 MHz, DMSO-d_6_): δ = 0.65 (d, ^3^*J*_HH_ = 6.6 Hz, 3H, CH_3_), 1.09 (d, ^3^*J*_HH_ = 6.6 Hz, 3H, CH_3_), 2.54–2.57 (m, 1H, CH(CH_3_)_2_), 6.34 (s, 1H, 1-CH), 7.45–7.47 (m, 1H, 6-CH), 7.53–7.54 (m, 1H, 8-CH), 7.70–7.74 (m, 1H, 9-CH), 8.53 (dd, 1H, 7-CH), 9.44 (s, 1H, OH); ^13^C-NMR (150.9 MHz, DMSO-d_6_): δ = 16.8 (s, CH_3_), 17.1 (s, CH_3_), 33.0 (s, CH(CH_3_)_2_), 90.3 (s, CH-3a), 115.2 (s, CH-9a), 117.1 (s, CH-8), 125.2 (s, C-6), 125.5 (s, CH-9), 133.2 (s, C-7), 136.1 (s, C-1a), 140.5 (s, CH-3), 155.5 (s, CH-1), 156.1 (s, CH-5a), 167.1 (s, C=O); MS *m/z* (%): 243 (M^+^) (11), 241 (72), 226 (54), 214 (100), 213 (56), 201 (45), 198 (78), 173 (52), 146 (28); Anal. Calcd. for C_14_H_1__3_NO_3_ (243.26): C 69.12; H 5.39; N 5.76%. Found: C 69.15; H 5.40; N 5.79%.

*3-(1-Hydroxyethylidene)-4-(nitromethyl)chroman-2-one *(**11**). Yield: 0.375 g (75%); colorless crystals, purified by recrystallization from ethanol, m.p. = 72.1–74.5 °C. IR (CHCl_3_): ν = 1750, 1600, 1450 cm^−1^; ^1^H-NMR (600 MHz, CDCl_3_): δ = 2.28 (s, 3H, CH_3_), 4.39 (dd, ^3^*J*_HH_ = 6.6 Hz, ^2^*J*_HH_ = 11.4 Hz, 1H, CH_2_NO_2_), 4.45 (dd, ^3^*J*_HH_ = 8.4 Hz, ^2^J_HH_ = 12.0 Hz, 1H, CH_2_NO_2_), 4.59 (t, ^3^*J*_HH_ = 7.2 Hz, ^3^*J*_HH_ = 7.2 Hz, 1H, 4-CH), 7.15–7.22 (m, 3H, 6-CH, 7-CH, 8-CH), 7.36–7.39 (m, 1H, 5-CH), 13.31 (s, 1H, OH); ^13^C-NMR (150.9 MHz, CDCl_3_): δ = 18.8 (s, CH_3_), 37.0 (s, CH-4), 80.5 (s, CH_2_NO_2_), 92.1 (s, C-3), 117.6 (s, CH-8), 120.5 (s, C-4a), 125.5 (s, C-6), 127.8 (s, CH-7), 130.0 (s, C-5), 150.3 (s, C-8a), 169.0 (s, C=O), 179.1 (s, C(OH)(CH_3_)); MS *m/z* (%): 249 (M^+^) (8), 188 (43), 173 (100), 145 (18), 118 (15), 105 (22); Anal. Calcd. for C_12_H_11_NO_5_ (249.22): C 57.83; H 4.45; N 5.62%. Found: C 57.80; H 4.40; N 5.63%.

*3-Acetyl-1-hydroxy-4-(2-hydroxyphenyl)pyrrolidine-2,5-dione *(**12**). Yield:0.055 g (11%); yellow crystals, purified by recrystallization from ethanol, m.p. = 76–79 °C. IR (nujol): ν = 3300, 1780, 1720, 1700, 1680, 1470, 1380 cm^−1^; ^1^H-NMR (250 MHz, DMSO-d_6_): 2.36 (s, 3H, CH_3_), 4.16 (d, ^3^*J*_HH_ = 4.5 Hz, 1H, 3-CH), 4.28 (d, ^3^*J*_HH_ = 4.3 Hz, 1H, 4-CH), 6.95–7.04 (m, 2H, 5′-CH, 3′-CH), 7.13–7.21 (m, 2H, 4′-CH, 6′-CH), 9.94 (s, 1H, OH), 10.77 (s, 1H, N-OH); ^13^C-NMR (62.9 MHz, DMSO-d_6_): δ = 25.6 (s, CH_3_), 42.4 (s, CH-4), 57.3 (s, CH-3), 115.2 (s, CH-5′), 119.0 (s, CH-3′), 123.0 (s, C-1′), 129.1 (s, CH-6′), 131.5 (s, C-4′), 155.2 (s, C-2′), 168.0 (s, C=O), 172.2 (s, C=O), 200.6 (s, COCH_3_). MS *m/z* (%): 249 (M+) (48), 231 (37), 206 (89), 190 (63), 173 (100), 147 (74), 146 (64), 118 (30), 91 (33), 77 (8); Anal. Calcd. for C_1__2_H_1__1_NO_5_ (249.22): C 57.83; H 4.45; N 5.62%. Found: C 57.81; H 4.43; N 5.63%.

*3-Nitro-4-(nitromethyl)chroman-2-one *(**13**). Yield: 0.454 g (90%); colorless crystals, purified by recrystallization from ethanol, m.p. = 83–84 °C. IR (nujol): ν = 1780, 1570, 1550, 1450, 1370, 1350 cm^−1^; ^1^H-NMR (600 MHz, DMSO-d_6_): δ = 4.8 (dd, ^3^*J*_HH_ = 5.1 Hz, ^3^*J*_HH_ = 5.4 Hz, 1H, 4-CH), 5.20 (dd, ^3^*J*_HH_ = 4.2 Hz, ^3^*J*_HH_ = 4.8 Hz, ^2^*J* = 15.0 Hz, 1H, CH_2_NO_2_), 5.34 (dd, ^3^*J*_HH_ = 4.8 Hz, ^3^*J*_HH_ = 5.4 Hz, ^2^*J* = 15.0 Hz, 1H, CH_2_NO_2_), 6.55 (d, ^3^*J* = 6.6 Hz, 1H, 3-CH), 7.24–7.31 (m, 2H, 8-CH, 6-CH), 7.44–7.53 (m, 2H, 7-CH, 5-CH); ^13^C-NMR (150.9 MHz, DMSO-d_6_): δ = 37.3 (s, CH-4), 75.0 (s, CH_2_NO_2_), 83.7 (s, CH-3), 117.1 (s, C-6), 117.5 (s, C-4a), 125.7 (s, CH-8), 128.0 (s, CH-5), 130.3 (s, CH-7), 149.7 (s, C-8a), 158.1 (s, C=O); MS *m/z* (%): 253 ((M+1)^+^) (6), 191 (53), 189 (24), 175 (22), 161 (98), 133 (100), 131 (48), 119 (42),115 (52), 111 (95), 109 (70); Anal. Calcd. for C_1__0_H_8_N_2_O_6_ (252.18): C 47.63; H 3.20; N 11.11%. Found: C 47.60; H 3.23; N 11.09%. 

*4-(Nitromethyl)-2-oxochroman-3-ylideneazinic acid* (**14**). It was obtained after 170 hours from compound **13** in DMSO solution; NMR monitoring was applied. Yield: 0.454 g (100%); yellow crystals, m.p. = 128–130 °C. IR (nujol): ν = 3320, 1740, 1620, 1550, 1450, 1370 cm^−1^; ^1^H-NMR (600 MHz, DMSO-d_6_): δ = 4.95 (t, ^3^*J*_HH_ = 4.2 Hz, 1H, 4-CH), 5.15 (dd, ^3^*J*_HH_ = 4.2 Hz, ^2^*J*_HH_ = 15.0 Hz, 1H, CH_2_NO_2_), 5.26 (dd, ^3^*J*_HH_ = 3.0 Hz, ^2^*J*_HH_ = 15.0 Hz, 1H, CH_2_NO_2_), 7.12–7.23 (m, 2H, 8-CH, 6-CH), 7.37–7.57 (m, 2H, 7-CH, 5-CH), 13.68 (s, 1H, OH); ^13^C-NMR (150.9 MHz, DMSO-d_6_): δ = 34.8 (s, CH-4), 78.2 (s, CH_2_NO_2_), 117.3 (s, CH-8), 119.2 (s, C-4a), 125.3 (s, C-6), 129.3 (s, CH-7), 130.0 (s, C-5), 144.6 (s, C-3), 150.4 (s, C-8a), 156.7 (s, C=O); MS *m/z* (%): 252 (M^+^) (11), 235 (15), 234 (35), 206 (76), 186 (47), 176 (48), 158 (37), 145 (70), 129 (100); Anal. Calcd. for C_1__0_H_8_N_2_O_6_ (252.18): C 47.63; H 3.20; N 11.11%. Found: C 47.66; H 3.23; N 11.08%. 

*2-Hydroxy-2,2a-dihydrochromeno[3,4-b]azet-3-one *(**16**). Yield: 0.209 g (55%); yellow crystals, purified by column chromatography on silica gel (hexane/ethyl acetate, 9:1 to 1:1), m.p. = 211–212 °C. IR (nujol): ν = 3300, 1710, 1680, 1600, 1560, 1450 cm^−1^; ^1^H-NMR (600 MHz, DMSO-d_6_): δ = 4.91 (s, 1H, 2a-CH), 6.47 (s, 1H, 1-CH), 6.85–6.89 (m, 2H, 5-CH, 7-CH), 7.48–7.52 (m, 2H, 6-CH, 8-CH), 13.06 (s, 1H, OH); ^13^C-NMR (150.9 MHz, DMSO-d_6_): 74.2 (s, CH-2a), 112.0 (s, CH-1a), 116.8 (s, CH-5), 118.1 (s, C-8a), 122.2 (s, CH-7), 124.4 (s, CH-1), 128.7 (s, CH-8), 132.0 (s, CH-6), 154.3 (s, C-4a), 161.6 (s, C=O); MS *m/z* (%): 189 (M^+^) (15), 171 (27), 149 (49), 143 (100), 129 (64), 121 (68); Anal. Calcd. for C_10_H_7_NO_3_ (189.17): C 63.49; H 3.73; N 7.40%. Found: C 63.44; H 3.69; N 7.45%.

*1-Hydroxy-3-(2-hydroxyphenyl)pyrrolidine-2,5-dione *(**17**). Yield: 0.083 g (21%); yellow oil, purified by column chromatography on silica gel (hexane/ethyl acetate, 9:1 to 1:1). IR (nujol): ν = 3400, 1620, 1640, 1450, 1370 cm^−1^; ^1^H-NMR (600 MHz, DMSO-d_6_): 2.56 (dd, ^3^*J*_HH_ = 4.8 Hz, ^2^*J*_HH_ = 17.4 Hz, 1H, 4-CH), 3.04 (dd, ^3^*J*_HH_ = 9.0 Hz, ^2^*J*_HH_ = 17.4 Hz, 2H, 4-CH), 4.27 (dd, ^3^*J*_HH_ = 4.8 Hz, ^3^*J*_HH_ = 9.0 Hz, 1H, 3-CH), 6.76–6.83 (m, 2H, 5′-CH, 3′-CH), 7.12–7.14 (m, 2H, 4′-CH, 6′-CH), 9.90 (s, 1H, OH); ^13^C-NMR (150.9 MHz, DMSO-d_6_): δ = 33.6 (s, CH-4), 40.8 (s, CH-3), 115.8 (s, CH-5′), 119.4 (s, CH-3′), 124.8 (s, C-1′), 129.3 (s, CH-6′), 131.5 (s, C-4′), 155.6 (s, C-2′), 172.5 (s, C=O), 174.5 (s, C=O); MS *m/z* (%): 207 (M^+^) (43), 190 (14), 178 (22),165 (23), 146 (49),135 (41), 121 (100); Anal. Calcd. for C_10_H_9_NO_4_ (207.18): C 57.97; H 4.38; N 6.76%. Found: C 57.95; H 4.38; N 6.75%.

Crystallographic data for the structures in this paper have been deposited in the Cambridge Crystallographic Data Center as a supplementary publication (**3**, CCDC 862726; **6**, CCDC 862727). Copies of the data can be obtained, free of charge, on application to CCDC, 12 Union Road, Cambridge CB12 1EZ, UK fax: +44-1223-336033 or e-mail: deposit@ccdc.cam.ac.uk.

## 4. Conclusions

In conclusion, a new stereospecific rearrangement reaction of 3-substituted coumarins was developed. It was established that coumarins which contain electron withdrawing groups gave the expected pyrrolidine derivatives. The exception is those compounds which prefer to generate stable enol forms of the products of addition of nitromethane by Michael reaction. Coumarins substituted with electron donating groups did not react at all. 2*H*-Chromen-2-one reacted under these conditions but for a longer reaction time and to give a low yield of the product. It is noteworthy that the thus synthesized heterocyclic compounds contain three pharmacophore fragments simultaneously, which might be interesting for biomedical investigations. Further applications of these processes to the preparation of targets of biological interest are under active investigation, and the results will be reported in due course.
